# Comparative effect of immunotherapy and standard therapy in patients with high grade glioma: a meta-analysis of published clinical trials

**DOI:** 10.1038/s41598-018-30296-x

**Published:** 2018-08-07

**Authors:** Stefan-Alexandru Artene, Adina Turcu-Stiolica, Marius Eugen Ciurea, Catalin Folcuti, Ligia Gabriela Tataranu, Oana Alexandru, Oana Stefana Purcaru, Daniela Elise Tache, Mihail Virgil Boldeanu, Cristian Silosi, Anica Dricu

**Affiliations:** 1 Unit of Biochemistry, University of Medicine and Pharmacy of Craiova, Craiova, Romania; 2 Department of Biostatistics, University of Medicine and Pharmacy of Craiova, Craiova, Romania; 3 Plastic Surgery Clinic, Emergency County Hospital of Craiova, Craiova, Romania; 4 Emergency Clinical Hospital “Bagdasar-Arseni”, Bucharest, Romania; 5 “Carol Davila” University of Medicine and Pharmacy, Bucharest, Romania; 6 Department of Neurology, University of Medicine and Pharmacy of Craiova, Craiova, Romania; 7 Department of Immunology, University of Medicine and Pharmacy of Craiova, Craiova, Romania; 8 Department of Surgery, University of Medicine and Pharmacy of Craiova, Craiova, Romania

## Abstract

Immunotherapy holds great promise in the treatment of high grade glioma (HGG). We performed a comprehensive meta-analysis of clinical trials involving dendritic cell (DC) therapy and viral therapy (VT) for the treatment of HGG, in order to assess their clinical impact in comparison to standard treatments in terms of overall survival (OS) and progression-free survival (PFS). To our knowledge, this is the first meta-analysis to evaluate VT for the treatment of HGG, allowing comparison of different immunotherapeutic approaches. Thirteen eligible studies of 1043 cases were included in the meta-analysis. For DC vaccination, in terms of OS, both newly diagnosed patients (HR, 0.65) and patients who suffered from recurrent HGGs (HR = 0.63) presented markedly improved results compared to the control groups. PFS was also improved (HR = 0.49) but was not statistically significant (p = 0.1). A slight improvement was observed for newly diagnosed patients receiving VT in terms of OS (HR = 0.88) while PFS was inferior for patients in the experimental arm (HR = 1.16). Our results show that DC therapy greatly improves OS for patients with both newly diagnosed and recurrent HGGs. VT, however, did not provide any statistically significant improvements in terms of OS and PFS for patients with newly diagnosed HGGs.

## Introduction

In light of the development of new therapeutic agents, many forms of cancer have seen a significant improvement in prognosis and overall quality of life. Despite this, advances in the treatment of brain cancers, more specifically high grade gliomas (HGG), which are the most common brain malignancies, have not yet yielded any noteworthy improvements. The current therapeutic standard of care, established in 2005, which involves surgical resection with adjuvant hyperfractionated radiotherapy and concomitant temozolomide (TMZ) still presents a poor outcome: overall survival (OS) is 14.6 months while progression-free survival (PFS) is 6.9 months^1^.

One of the main reasons for the poor outcome is the body’s limited immune response directed against brain tumors. Initially, this was attributed to the existence of the Blood Brain Barrier which granted the Central Nervous System (CNS) an immune-privileged status. However, recent discoveries have disproven this notion^2–4^. Even in the presence of immune cells in the tumor microenvironment, HGGs still successfully hinder the body’s capacity to specifically target and destroy tumor cells. Additionally, an increased immune presence in the tumor does not translate into an improved prognosis^5^. This increased evasive capacity of HGG in the face of an immune response is due to a number of factors. Most notably, HGGs have been shown to actively downregulate the expression of the major histocompatibility complex class I (MHC I) and class II (MHC II) on the surface of microglial cells^6^. This, coupled with the increased production of immunomodulatory cytokines such as Tumor growth factor β (TGF β), prostaglandin E and Interleukins 4, 6 and 10 (IL-4, IL-6 and IL-10) have a detrimental effect on the immune modulating activity of microglias^7^. Furthermore, active recruitment and accumulation of regulatory T-Cell (T-regs) in the tumor expressing higher levels of Programmed Death-1(PD-1) and Cytotoxic T-lymphocyte Antigen-4 (CTLA-4) than peripheric T-regs further serves to highlight the strong immunosuppressive effect of the tumoral microenvironment^8,9^.

Given the abundance of interactions between cancer cells and the immune system, through which the former evades the action of the latter, approaches that make the tumor vulnerable to immune attacks have always been considered as a strong candidate. DCs play a detrimental role in eliciting an immune response against foreign antigens, acting as mediators between the adaptive and the innate immune systems. Their main role is to recognize and take up antigens, process and present them to either T-cells or B-cells, thus triggering a cell-based immune response or dampening any immune reactivity towards self-antigens^10^. DCs are seen as having the highest capacity to trigger a T-cell or B-cell *de novo* immune response against a foreign antigen, making them a highly attractive candidate for developing vaccines capable of eliciting an immune response against tumor cells^11^. For DCs to trigger an immune response they must go through a series of procedures. First, they must be differentiated from patients’ peripheral blood mononuclear blood cells^12,13^ after which they must be matured using a cytokine cocktail^14,15^. In order for DCs to be able to recognize tumors cell from the surrounding tissue, they must be loaded with antigens which are specific to the malign cells. These antigens can be either tumor specific antigens (TSAs) or tumor associated antigens (TAA). DC vaccination is the oldest form of immunotherapy used in the treatment of HGGs with the first phase I trial being conducted in 2001 by Yu *et al*. Viruses are organisms capable of binding to a specific cell type, hijacking the cells replicative mechanisms in order to bolster its own numbers leading to the host’s death and release of viral copies which move on to invade more cells. This capacity to selectively attack and overwhelm a certain cellular population while completely ignoring the other cells in its proximity has made VT an attractive option in cancer treatment. The most popular VT used in the treatment of HGG is suicide gene therapy, where a viral vector inserts a gene into the host’s genome, forcing the gene to synthesize an enzyme capable of transforming an inactive prodrug into an active agent which completely blocks cellular replication^16^. Given their propensity for invading neural structures, herpes simplex viruses have been extensively used to deliver the thymidine kinase (HSV-Tk) gene which converts the prodrug ganciclovir into ganciclovir triphosphate, resulting into cell cycle arrest and subsequent apoptosis^17^. Another viral-based approach involves the use of oncolytic agents. Direct agents selectively target tumor-specific structures^18,19^ while indirect viral agents aim to enhance antitumor immunity by infecting cancer cells and triggering an adaptive immune system response against the host cells^20^.

While immunotherapy has proven to be very popular in other cancers, it failed to reach the same popularity in treating HGG. The aim of our study is to compare the effect of the two most popular immune therapies which are currently being tested in clinical trials, to standard of care treatments. The aim is to assess if these approaches can be considered as viable alternatives or even possible future replacements for the current standard of care treatments which are considered disappointing in terms of outcome and quality of life^21^. Therefore, we performed a comprehensive meta-analysis of clinical trials involving immunotherapy with either DCs or VT for the treatment of GBM.

## Results

### Trial selection

**Figure 1:**
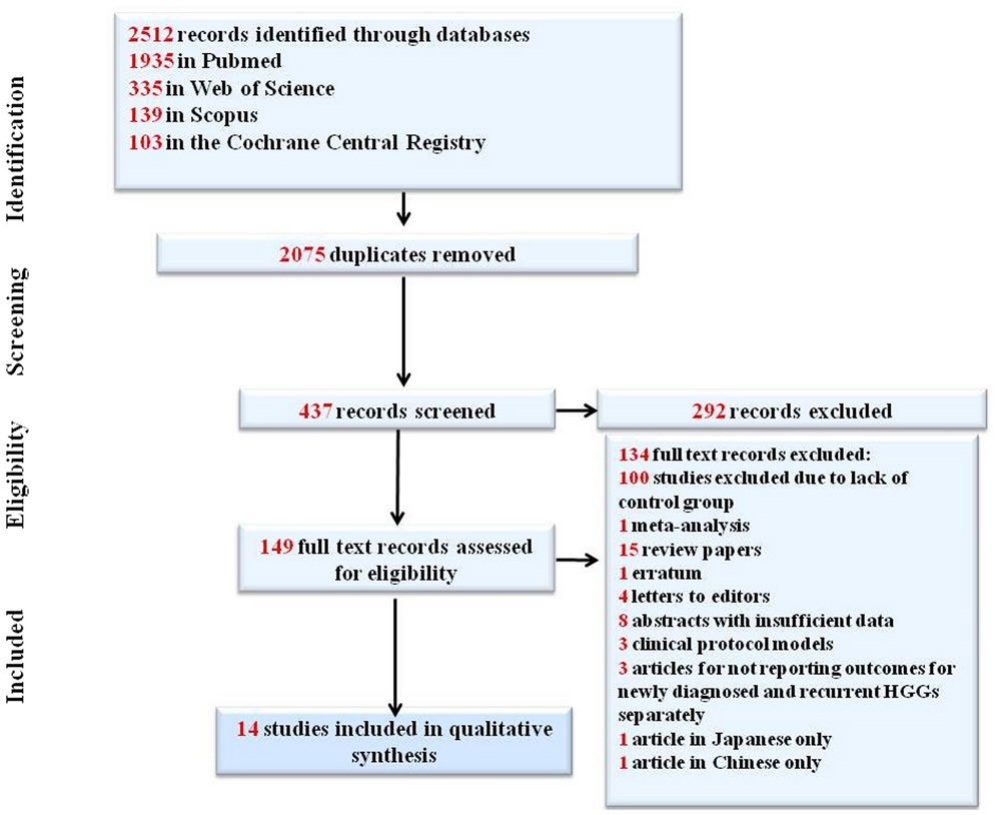
PRISMA flow diagram.

The electronic search yielded 2512 results combined between all databases (1935 in Pubmed, 335 in Web of Science, 139 in Scopus and 103 in Cochrane Central Registry). Of these, 2075 of the results were excluded as duplicates. A title and abstract review was performed in the remaining 437 articles resulting in the exclusion of 292 results. We retrieved the full text of the remaining 149 articles and 133 were excluded for the following reasons (Fig. 1). 14 studies were initially selected for inclusion in our study: 9 with patients receiving DC therapy, 4 with patients receiving viral therapy and only 1 where patients received adoptive therapy^22^. No studies were identified at the time of the search for patients using peptide vaccines and checkpoint inhibitors that met the inclusion criteria. Because of the discrepancy between the relatively large number of studies in the DC and VT arm (9 for DC therapy and 4 for VT) and the adoptive therapy arm where only 1 study was available we opted to exclude the adoptive therapy study and discuss it separately. Due to the different prognosis of newly diagnosed and relapsed HGGs, the studies were divided into subgroups in accordance to this criterion. For patients receiving DC vaccination, 6 studies reporting OS^23–28^ and 2 studies reporting PFS^25,27^ were included in the newly diagnosed subgroup while 3 studies reporting OS^24,29,30^ were included in the recurrent subgroup. For VT therapy, 4 studies reporting OS^31–34^ and 3 studies reporting PFS^31,33,34^ were included in newly diagnosed subgroup. Due to an insufficient number of studies, we could not carry a meta-analysis on PFS for patient suffering from recurrent HGGs who received DC vaccination and a meta-analysis of OS and PFS for patients suffering from recurrent HGGs who received VT.

### Meta-analysis of Overall Survival and Progression-free survival in dendritic cell vaccination studies

**Figure 2:**
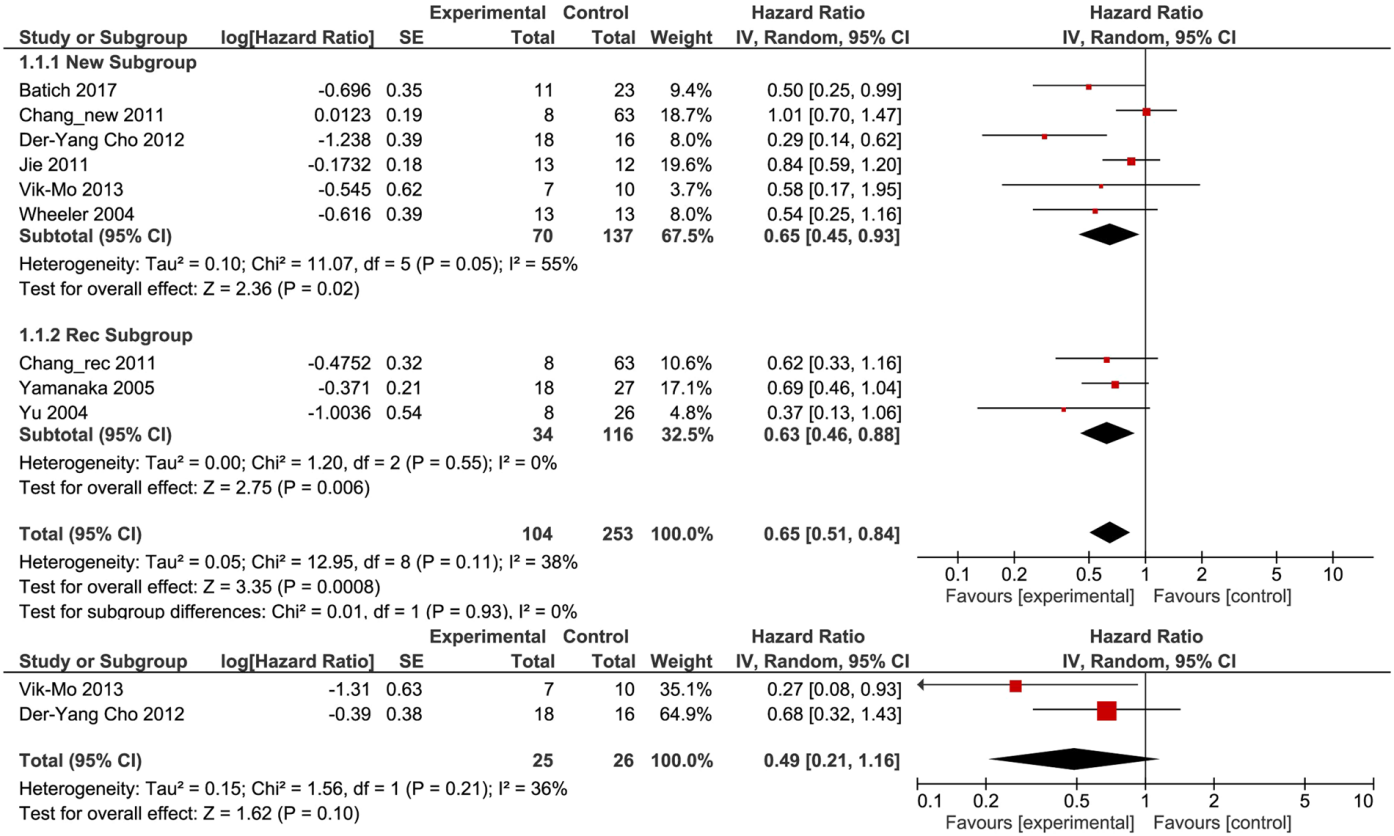
HR (Hazard Ratio) for OS (**a**) and PFS (**b**) for patients receiving DC vaccination versus standard therapy. The random effects model (Mantel-Haenszel method) was used for the analysis. HR = hazard ratio OS = overall survival DC = dendritic cell.

Nine studies with a total of 357 patients (104 patients in Experimental arms and 253 patients in Control arms) were included in the meta-analysis of DC treatment (Table 1). The studies were divided into two subgroups: one subgroup of 207 newly diagnosed patients (70 patients in the experimental arm and 137 in the control arm) and one subgroup with 150 patients diagnosed with recurrent tumors (34 patients in the experimental arm and 116 in the control arm). Because of different sample sizes, each study has a different level of sampling error (SE). In total, nine studies were included in a meta-analysis of OS (6 for newly diagnosed patients and 3 for patients with recurrent tumors) and two studies were included in a meta-analysis of PFS for newly diagnosed patients receiving DC-based therapy. We applied random effects to our analysis. The weight of random effects meta-analyses is relatively more equal than fixed effect analyses by incorporating the study variance into the denominator of each weight. In terms of OS, each DC treatment had a stronger effect in comparison to the control arm, with the most significant results being reported by Der-Yang Cho *et al*.^25^ (HR, 0.29, 95% CI: 0.14–0.62) while the weakest correlation between DC treatment and OS was reported by Chang *et al*.^24^ (HR, 1.01, 95% CI: 0.70–1.47) (Fig. 2a). Thus, the variability across effect sizes does not overtake what would be expected based on SE. The summary effect of our meta-analysis on OS for studies regarding DC therapy indicates a major improvement in OS for both newly diagnosed (HR, 0.65, 95% CI: 0.45–0.93) and patients suffering from recurrent HGGs (HR, 0.63, 95% CI: 0.46–0.88) in the experimental arm in comparison to those in the control group. No evidence was found for any differences between the subgroups of newly diagnosed patients and patients diagnosed with recurrent tumors (p = 0.93). The value of the ratio of true heterogeneity to total variance across the observed effect estimates, *I*^2^, indicates low heterogeneity (38%) (Fig. 2a).

**Table 1:** Main characteristics of studies that use DC therapy for the treatment of HGGs.

Trial reference	Year	WHO tumor grade, histology and characteristics	Number of patients		Clinical trial phase	DC regimen	Endpoints
			Experimental arm	Control arm			
Wheeler *et al*.^28^	2004	IV(GBM), ND	13	13	IA/IB/II	Three vaccines containing 10–40 × 10^6^ DCs per unit, 2 weeks after surgery. A fourth vaccine was administered for patients in the vaccine-only arm.	OS,
Yu *et al*.^30^	2004	III(AA), IV(GBM), ND + REC	8	26	I	Three intradermal vaccines containing 10^7^–10^8^ DCs per unit at 2 weeks intervals.	OS
Yamanaka *et al*.^29^	2005	IV(GBM), REC	18	27	I/II	Up to twenty two intradermal (mean 7.4) and/or intratumoral (mean 4.6) vaccines, every three weeks, depending on clinical response. The mean number of DCs administered was 5.318 × 10^7^ cells for intradermal vaccines and 4.235 × 10^7^ cells for intratumoral vaccines.	OS
Chang *et al*.^24^	2011	III(AA, AO), IV(GBM), ND + REC	16	63	I/II	Up to ten subcutaneous vaccines containing 1.10–6.1 × 10^7^ DCs per unit, every week 4 times, every two weeks twice and every month 4 times.	OS
Jie *et al*.^26^	2011	IV(GBM), ND	13	12	I/II	Four subcutaneous vaccines containing 6 × 10^6^ DCs per unit at days 7, 14, 28 and 42. (day 0 is the day blood was drawn, 2 weeks after surgical resection)	OS
Der-Yang Cho *et al*.^25^	2012	IV(GBM), ND	18	16	II	Ten subcutaneous vaccines containing 2 × 10^7^ DCs per unit, every week for the first month, every two weeks for the next month and every month 4 times.	OS, PFS
Vik-Mo *et al*.^27^	2013	IV(GBM), ND	7	10	I/II	Two vaccines containing 10^7^ DCs per unit, during the first week after completing standard RT + CT regimen, one vaccine weekly for the next three weeks and adjuvant TMZ or DC vaccine every other week.	OS, PFS
Batich *et al*.^23^	2017	IV(GBM), ND	11	23	I	One DC vaccine each month, on day 23 ± 1 of a 28 day cycle, alongside a dose-intense TMZ regimen from days 1–21 of the cycle, monthly, until disease progression.	OS

GBM-Glioblastoma, AA-anaplastic astrocytoma, AO-anaplastic oligodendroglima, ND: newly diagnosed; REC-Recurrent, RT-radiotherapy, CT-chemotherapy, TMZ-Temozolomide.

Three studies with 51 patients (25 patients in experimental arms and 26 patients in control arms) were included in the meta-analysis of PFS for patients with newly diagnosed HGGs (Fig. 2b). As observed in Fig. 2b, all the studies yielded improvements in PFS, with the strongest effect being reported by Vik-Mo *et al*.^27^ (HR 0.27; 95% CI: 0.08–0.93). Combined, DC treatment presents an improved PFS (HR = 0.49), but with a very large 95% CI (0.21–1.16). An *I*^2^ of 36% indicates low heterogeneity while a Chi^2^ of 1.56, which is smaller than (χ^2^_0.05_ for df = 1), indicates the failure to reject the null hypothesis of homogeneity. The overall results in the case of PFS for DC treatment were marginally better, but not statistically significant (Z = 1.62, p = 0.10). Given the relative small number of studies involved (2) and moderate heterogeneity (I^2^ = 36%), we can conclude that almost all of the observed variance is spurious, incorporating both true heterogeneity and also random error (Fig. 2b).

### Publication bias

**Figure 3:**
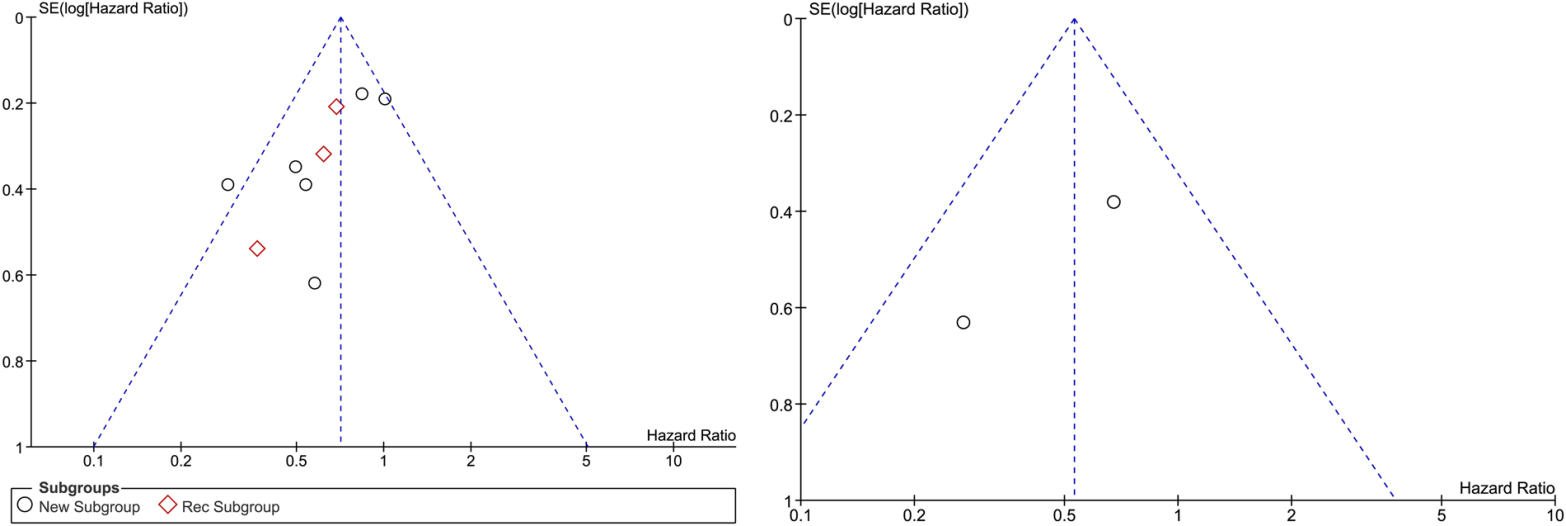
Funnel plot presenting the association between DC (**a**) and PFS (**b**) therapy and OS.

The visual inspection indicated a possibility of publication bias due to the slight asymmetry of the funnel plots for OS and PFS (Fig. 3). The relationship between study size and effect size is displayed by the funnel plots with a notable absence of smaller studies toward the bottom of the graph. Bias against the publication of small studies with null results or results showing an adverse effect of the treatment is presented as the absence of corners in the funnel plot. However, we can observe the symmetrical distribution of the studies around the mean effect size which indicates a relative absence of publication bias.

### Meta-analysis of Overall Survival and Progression-free survival in Viral Therapies studies

Four studies with 642 newly diagnosed patients (237 patients in the experimental arm and 405 patients in the control arm) were included in the meta-analysis of VT. In total, four studies were included in the meta-analysis of OS and three studies were included in the meta-analysis of PFS (Table 2).

**Table 2:** Main characteristics of studies that use VT for the treatment of HGGs.

Trial reference	Year	WHO tumor grade, histology and characteristics	Number of patients		Clinical trial phase	VT regimen	Endpoints
			Experimental arm	Control arm			
Rainov *et al*.^34^	2000	IV(GBM), ND	111	103	III	Intracranial injection of HSV-tk VPC immediately after surgical resection (day 0). Ganciclovir was given i.v. on days 14 to 27 in two daily 5 mg/kg doses.	OS, PFS
Westphal *et al*.^32^	2013	IV(GBM), ND	119	117	III	Intracranial injection of Sitimagene Ceradovec® immediately after surgical resection (day 0). Ganciclovir was given i.v. on days 5 to 18 in two daily 5 mg/kg doses.	OS
Stragliatto *et al*.^33^	2013	IV(GBM), ND	22	20	I/II	Two 450 mg Valganciclovir tablets twice daily from weeks 0–3. One 450 mg Valgaciclovir tablet twice daily weeks 4–24.	OS, PFS
Wheeler *et al*.^31^	2016	III(AA, AO), IV(GBM), ND	48	134	Ib/IIb	12 patients received 3 × 10^10^, 1 × 10^11^, and 3 × 10^11^AdV-tk vector particles in the initial Ib trial; 36 patients received 3 × 10^11^AdV-tk vector particles in the IIb trial; Valacyclovir was given orally on days 1–3 to 14–16 in three daily 3 g doses (i.v. acyclovir 10 mg/kg for patients who were unable to take oral medication)	OS,PFS

GBM-Glioblastoma, AA-anaplastic astrocytoma, AO-anaplastic oligodendroglima, ND: newly diagnosed; REC-Recurrent, HSV-tk-Herpes Simplex Virus-thymidine kinase, ADV-tk: replication-deficient adenovirus mutant thymidine kinase, AdV-tk-aglatimagene besadenovec, RT-radiotherapy, CT-chemotherapy; VT-Viral Therapy, PFU-plaque-forming unit.

**Figure 4:**
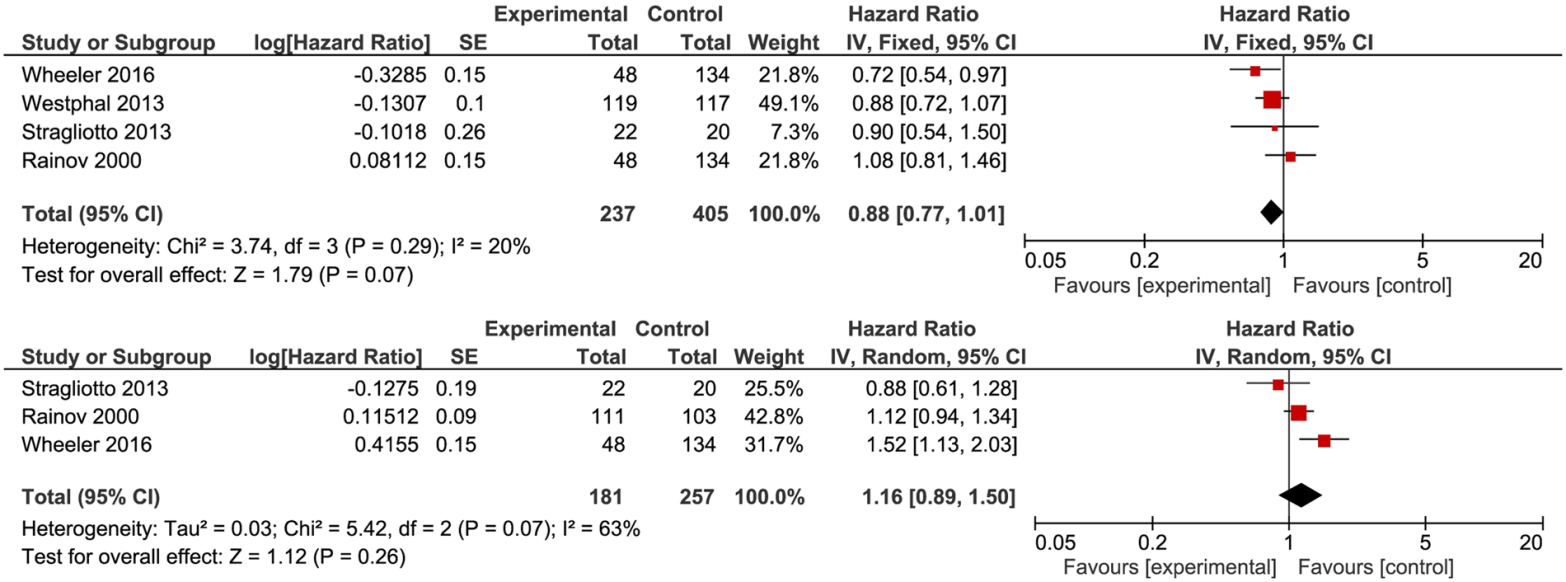
HR (Hazard Ratio) for OS (**a**) and PFS (**b**) for patients receiving VT versus standard therapy. The random effects model (Mantel-Haenszel method) was used for the analysis. HR = hazard ratio OS = overall survival VT = viral therapy.

As seen in Fig. 4a, three of the four studies reported improved OS for patients receiving VT, with the most notable effect being reported by Wheeler *et al*.^31^ (HR 0.72, 95% CI: 0.54–0.97). Rainov *et al*.^34^ reported a marginally better outcome for patients in the control arm (HR 1.08, 95% CI: 0.81–1.46) (Fig. 4a). Overall, by using a random-effect model, a meta-analysis of OS demonstrated a slightly improved outcome for newly diagnosed patients who received VT (HR, 0.88, 95% CI 0.75–1.04). The test for homogeneity (Chi^2^ = 3.74 < 7.81, χ^2^_0.05_ for df = 3, p = 0.29) does not reject the null hypothesis of homogeneity, with all of the effect size not being an estimate of the same population mean. *I*^2^ was 20% indicating low heterogeneity (Fig. 4a). The test for overall effect did not provide a significant statistical (Z = 1.52, p = 0.13) association between VT and improved outcome in terms of OS (Fig. 4a).

Three studies with 438 patients (181 patients in Experimental arms and 257 patients in Control arms) were included in the meta-analysis of PFS (Fig. 4b).The effect of VT for patients with newly diagnosed HGGs was inferior to standard treatment protocols (HR = 1.16, 95% CI 0.89–1.50). Additionally, the Z-value is 1.12 < 1.96 (corresponding to the two-tailed alpha of 0.05) and p = 0.26 (p > 0.05), which allows the use of the null hypothesis of no differences between the two groups of patients and to conclude that the effect is not statistically significant. The value of *I*^2^ was 63%, translating as high heterogeneity, which means that the observed variance comes from real differences between studies and therefore, can potentially be explained by study-level covariates (Fig. 4b).

**Figure 5:**
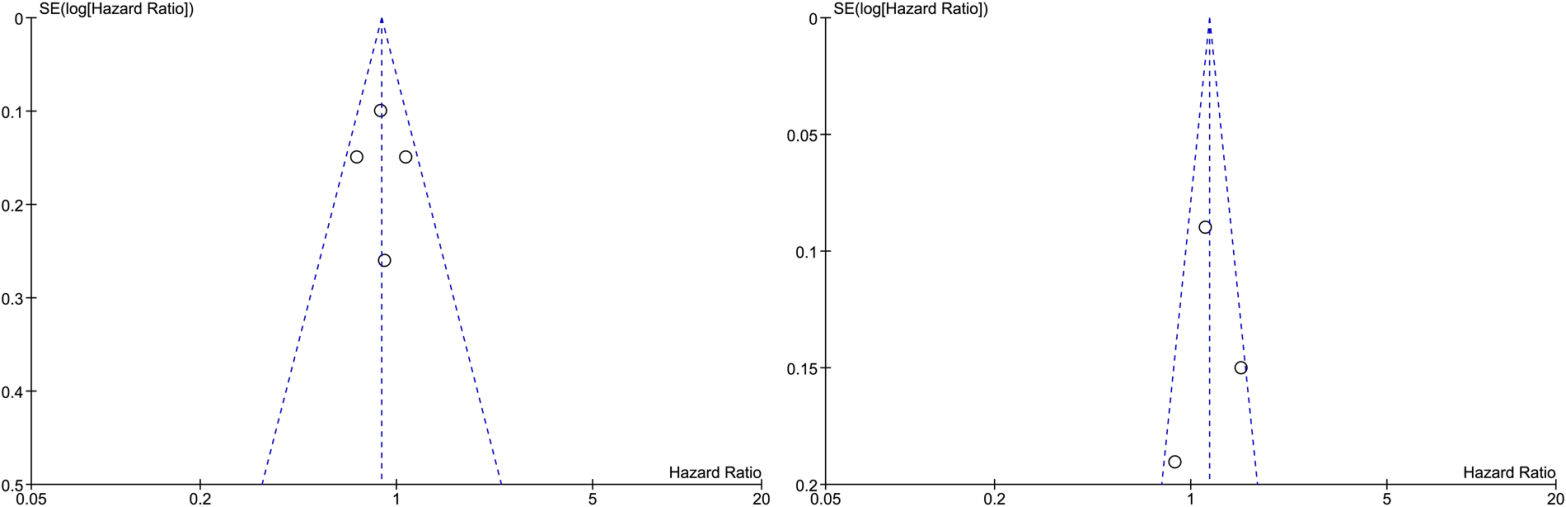
Funnel plot presenting the association between VT therapy and OS (**a**) and PFS (**b**).

The subjective impression from the funnel plots (Fig. 5) does support the presence of publication bias: symmetry at the top, some studies missing from the bottom of the graph and one study is outside of the funnel. However, studies with larger than average effects are more likely to be published, and this could be the reason of the upward bias in the summary effect.

Overall, our study demonstrated that DC therapy improves OS for patients with newly diagnosed HGGs and for patients suffering from recurrent HGGs. In terms of OS, DC therapy presented significantly better results for patients with newly diagnosed HGGs, with patients being 35% more likely to have an improved OS (HR = 0.65, 95% CI: 0.45–0.93, p = 0.02) in comparison to those receiving standard therapy. For patients with relapsed HGGs, DC vaccination improved OS by a similar margin (HR, 0.63, 95% CI: 0.46–0.88, p = 0.006). In addition, heterogeneity between studies was low (I^2^ = 38%) thus further solidifying the statistical significance of our study.

Regarding overall PFS, newly diagnosed patients receiving DC treatment presented a 51% increased chance (HR, 0.49, 95% CI: 0.21–1.16, p = 0.1) of experiencing a prolonged period between the initiation of treatment and MRI confirmed tumor recurrence. However, the results were not statistically significant (p = 0.10) with very large confidence intervals being observed (0.21–1.16) mainly due to the small number of patients included in the experimental arm. Low heterogeneity was observed (I^2^ = 36%) between the studies included in the PFS analysis. Overall, our study showed that DC therapy significantly improves OS for patients suffering from both newly diagnosed and recurrent HGGs providing further justification for the development of larger studies based on this therapeutic approach. Visual analysis of the funnel plot of OS and PFS across trials with DC therapy has indicated possible publication bias, with studies being distributed relatively symmetrically around the mean effect size.

Our meta-analysis also showed improved OS for newly diagnosed patients receiving VT in comparison to those in the standard therapy arm. In regards to OS, patients undergoing VT were 28% more likely to experience an improvement (HR 0.72, 95% CI: 0.54–0.97) as opposed to those receiving standard treatment. However, the results were not statistically significant (p = 0.13) while heterogeneity between studies was low (I = 20%) confirmed by the x^2^ test (Chi^2^ = 3.74 < 7.81, χ^2^_0.05_ for df = 3, p = 0.29). In terms of PFS, patients receiving VT observed no improvement (HR = 1.16, 95% CI 0.89–1.50) in comparison to the patients in the control group. Additionally, we concluded that these results were not statistically significant (p = 0.26). Heterogeneity was also high (I^2^ = 63%). Visual analysis of funnel plots also supports the presence of publication bias.

When compared, DC treatment presents superior results in contrast to VT in terms of OS for patients with newly diagnosed HGGs. (35% improvement versus 12% for patients undergoing VT).

According to these results, DC vaccines represent a superior option to VT in terms of OS for patients with newly diagnosed HGGs. We could not make the same comparison for patients with recurrent HGGs due to an insufficient number of studies using VT for this specific group. We couldn’t extract the necessary information from the studies in order for us to perform a multivariate meta-regression. We have performed a sensitivity analysis by excluding the studies with smaller sample sizes in order to assess the stability of our results. The results obtained after exclusion did not vary from the original ones.

## Discussion

Even with the current therapeutic standard, GBM is considered an incurable disease with a poor prognosis and very limited treatment options for both newly diagnosed and recurrent tumors. The development of new therapeutic options for the treatment of GBM is imperative for improving life expectancy and overall quality of life for patients suffering from this form of cancer. The highest expectations are for newly diagnosed patients, as they receive the greatest potential benefit from novel treatments. While some treatment protocols such as bevacizumab alongside irinotecan have been established as being viable salvage options for the treatment of recurrent GBM, no new treatments for newly diagnosed tumors have been included into standard practice. However, a few recent meta-analysis and systematic reviews on targeted treatment for newly diagnosed GBM have indicated that combination between standard of care treatment and targeted agents could prove superior to standard of care treatments alone^35,36^. Both these studies present major limitations and the data presented is not sufficient for the combinations to be considered as strong candidates for inclusion into mainstay protocols. Immunotherapy has not fared better in treating newly diagnosed GBM patients, either. Vaccination with the agent Rindopepimut which triggers an immune reaction against a specific EGFR mutation (EGFRvIII) showed promising results in phase I and II clinical trials. Unfortunately, the phase III trial (ACT IV) showed no benefit for newly diagnosed patients in terms of OS. The results were initially retracted and republished in October 2017^37^.

DC vaccination has been considered as an important step in cancer treatment, although the results published over the years are not in perfect harmony causing uncertainty in making clinical decisions. Only one meta-analysis has, so far, analyzed the clinical and biological implications of DC treatment for patients with HGG^38^. The author showed that DC treatment markedly improves both OS and PFS for patients with HGG in comparison to those receiving standard treatment. In addition, peripheral high levels of IFN-γ corresponding to an elevated immune response were observed. In our previous study based on a survival gain analysis of patients receiving DC-based treatment versus bevacizumab and irinotecan for patients with recurrent HGG, we didn’t find any major improvement for both OS and weighted survival gain for patients in the immunotherapy arm^39^. However, it should be noted that we only included patients with recurrent tumors in the study, in which case treatment response rates, OS and PFS are significantly lower than in newly diagnosed patients.

In the present study, we demonstrated that newly diagnosed (HR, 0.65, 95% CI: 0.45–0.93 p = 0.02) and patients with recurrent HGGs (HR, 0.63, 95% CI: 0.46–0.88, p = 0.006) in the DC-based experimental arm presented significantly better OS while the improvement in PFS (HR, 0.49, 95% CI: 0.21–1.16) was not statistically significant (p = 0.1). Heterogeneity between studies was also low (I^2^ = 38%).

VT is based on the principle of a viral agent selectively infecting and destroying cancer cells through different means while leaving normal cells relatively untouched. While the idea originated almost a century ago, real progress in the field has only been made in the past two decades. Some viruses such as Herpes Simplex Virus-1 (HSV)^40,41^, Adenovirus^42^, Measles virus (NCT02962167) or Newcastle Disease Virus^43^ were used as direct oncolytic agents, while others were used as vectors for gene therapy^34,44,45^ making them susceptible to anti-viral prodrugs. Some authors have based their study on the presumption that gliomagenesis is linked to cytomegalovirus (CMV) infection^46,47^, treating CMV-positive GBM patients with valganciclovir^33^. No systematic analysis or meta-analysis of different VT protocols has been performed so far, in order to assess its efficacy compared to treatment currently used in standard clinical practice. To our knowledge, this is the first report to date for which a systematic review and meta-analysis of VT for the treatment of HGG has been performed and also where different immunotherapeutic approaches have been compared. In our study, newly diagnosed patients in the experimental VT arm presented improved OS (HR 0.72, 95% CI: 0.54–0.97) (p = 0.13) in comparison to patients in the control arm, with low heterogeneity between studies (I^2^ = 20%). Additionally, patients in the experimental VT arm experienced inferior PFS when compared to those in the control group (HR = 1.16, 95% CI 0.89–1.50) but the results were not statistically significant (p = 0.26) as well. However, these findings must be interpreted with caution, while taking into consideration several study limitations. First, there are major differences between the DC and VT arms in terms of number of patients, age, sex, performance status, comorbidities, adjuvant therapy after resection or concomitant therapy during immunotherapy. Additionally, some studies report patient outcomes without any distinction between grade III and grade IV HGGs^24,30,31^. The genetic and epigenetic differences between grade III and IV HGG such as O-6-methylguanine-DNA methyltransferase (MGMT) methylation status for GBM patients have a significant impact on treatment response and prognosis. Even amongst GBM patients, the presence of four subtypes with specific genetic characteristics can influence the results presented in clinical trials. Conclusions drawn from heterogeneous studies involving both grade III and IV HGGs should be met with great caution and considered of inferior statistical value when compared to larger, more specific studies involving only de novo or recurrent GBMs. Second, the lack of large, multicenter, randomized, double-blind phase 3 trials, which are considered of great value in terms of statistical outcomes, can heavily contribute to some of the imprecise or highly variable data we obtained in our study, such as large confidence intervals or high heterogeneity between studies. The majority of the studies used in our meta-analysis, especially in the DC cohort, are phase I/II studies with a very small number of patients that have limited statistical value. Additionally, the vast majority of studies included non-randomized or historical control groups, which further reduces the statistical significance of each individual study, potentially leading to an overestimation of the effect for each treatment option. Furthermore, most of the numbers obtained in our analysis are not derived from individual patient data, so the information presented could be independently verified by an external evaluator. Third, both DC and VT arms have a very high variability in terms of study design, including the nature of the therapeutic agent (mostly in the case of VT), preparation, administration and follow-up. Fourth, there are more immunotherapeutic options available at the moment than those discussed in our study. However, most of them are ongoing or are phase I/II clinical trials without a control arm. Only one phase III study published in 2016, with patients receiving adoptive therapy versus standard of care, is currently available^22^. This phase III study showed an improved PFS for patients in the experimental arm but similar OS between the experimental and control groups. Further studies are required for a more extensive analysis of different immune therapies and to demonstrate whether immunotherapy can become an option for adjuvant treatment of HGG. In our study we have shown that DC vaccination improves OS for both newly diagnosed and recurrent HGGs when compared to standard treatment while the improvement in PFS was not statistically significant for newly diagnosed patients. VT did not provide any statistically significant improvement to OS or PFS. However, no definitive conclusion can be drawn, as of yet, on the efficacy of both VT or DC vaccination in the treatment of HGG given that most studies in our meta-analysis are phase I/II clinical trials with limited statistical value. Prospective phase III clinical trials with large numbers of patients on either de novo or relapsed grade III or grade III tumors are required for a more definitive conclusion to be drawn.

## Materials and Methods

### Search strategy

An electronic search was performed in October 2016 and revised in December 2017, through Pubmed, Web of Science, Scopus, Embase and Cochrane Central Registry of Controlled Trials. The following keywords were used to screen for results: (“malignant glioma” or “high grade glioma” or “glioblastoma”) AND (“immunotherapy” OR “immune therapy”).

After the initial results, we expanded our search to similar articles and references mentioned in retrieved results. There were no limitations in regards to publication year. Only articles published in English were included in the screening process. Studies regarding non-human glioma models were excluded from the screening process. Studies that were not published as full reports, erratums and letters to editors were excluded from further statistical analysis.

### Study selection and definition of outcomes

Two independent authors (S.A. and A.T.) separately reviewed the publications resulted from our literature search. The following criteria were applied when the study selection was performed: 1) adult patients with histopathologic confirmation of HGG (grade 3 and 4 malignant gliomas) 2) studies with at least 2 distinct groups: one group where immunotherapy was performed without any conventional treatment (surgery and adjuvant chemo and radiotherapy) for HGG and one control group where patients received only conventional treatment 3) studies who reported outcomes for either newly diagnosed patients or patients suffering from recurrent HGGs as distinct entities. No restrictions were applied on the protocols used for immunotherapy or conventional treatment of GBM in both groups. No restrictions were applied on the number of patients included in each group or the nature of the control group used in the studies. The main outcome was overall survival (OS), while the secondary outcome analyzed in our study was progression-free survival (PFS). OS was defined as time from the initiation of treatment to the patient’s death by any cause. PFS was defined as the time between the initiation of treatment and disease progression confirmed by MRI scanning.

### Statistical analysis

Our analysis is based on a fixed effects model, because the inference is conditional and directly proportional to the number of studies involved in the meta-analysis. Very few clinical trials with control groups, receiving conventional treatment, have been conducted for new treatments of HGG. Even though the random effects model produces more results relying on the assumption that the true effect size varies between studies, in the case of a reduced number of studies, the between-studies variance estimate can be untrustworthy^48^. Time-to-event outcomes were analyzed using hazard ratios (HRs). For each study, the HR and its 95% confidence interval (95% CI) were recovered using a fixed inverse variance model. If these parameters were not available in the studies, we used WebPlot Digitizer (Austin, Texas, USA) to extract the specific survival rates according to the Kaplan-Meier curves and to calculate the HR by the methods described by Tierney *et al*.^49^. The analytical models were carried out using SPSS Statistics 20 (IBM Corp, Armonk, NY, USA). For each study, we extracted the number of patients from the experimental groups and control groups who experienced the events of interest.

Meta-analysis was performed for OS and PFS. The heterogeneity between studies was assessed by χ^2^test with p-value and *I*^2^ statistics (the percentage of the total variation in the overall results that is due to heterogeneity rather than chance) according to the method described by Higgins *et al*.^50^ and Borestein *et al*.^48^. P_heterogeneity_ < 0.1 or I^2^ > 50% are considered to be statistical significant. The χ^2^ test is used to analyze study heterogeneity by attempting to reject the null hypothesis that the studies are homogenous. A low p-value (<0.1) or a large chi-squared statistic relative to its degree of freedom (equal to the number of studies minus one) provides evidence of heterogeneity (variation in effect estimates beyond chance) and is considered statistically significant. An I^2^ > 50% is considered proof of heterogeneity. Publication bias of studies involved in our meta-analysis study was based on the visual inspection of the symmetry of funnel plots.

Forest plots were generated using Review Manager Software 5.0 (Nordic Cochran Centre, Copenhagen, Denmark) to illustrate results of individual studies and meta-analysis. P < 0.05 was considered statistically significant. An HR of 1.0 would mean that the risk of death was the same in both samples, while an HR less than 1.0 would mean that the risk was lower in the experimental group, and an HR greater than 1.0 would mean that the risk was lower in the control sample.

Larger weight (usually those with narrower confidence intervals) dominated the calculation of the pooled result. The confidence intervals describe the range of intervention effects compatible with the study’s result and indicate whether each was individually statistically significant.

The datasets generated and/or analyzed during the current study are available from the corresponding author on reasonable request.

## References

[1] Stupp R (2005). Radiotherapy plus concomitant and adjuvant temozolomide for glioblastoma. The New England journal of medicine.

[2] Carson MJ, Doose JM, Melchior B, Schmid CD, Ploix CC (2006). CNS immune privilege: hiding in plain sight. Immunological reviews.

[3] Hickey WF, Hsu BL, Kimura H (1991). T-lymphocyte entry into the central nervous system. Journal of neuroscience research.

[4] Laman JD, Weller RO (2013). Drainage of cells and soluble antigen from the CNS to regional lymph nodes. Journal of neuroimmune pharmacology: the official journal of the Society on NeuroImmune Pharmacology.

[5] Safdari H, Hochberg FH, Richardson EP (1985). Prognostic value of round cell (lymphocyte) infiltration in malignant gliomas. Surgical neurology.

[6] Zagzag D (2005). Downregulation of major histocompatibility complex antigens in invading glioma cells: stealth invasion of the brain. Laboratory investigation; a journal of technical methods and pathology.

[7] Cai J (2015). Identification of a 6-cytokine prognostic signature in patients with primary glioblastoma harboring M2 microglia/macrophage phenotype relevance. PloS one.

[8] Jacobs JF (2009). Regulatory T cells and the PD-L1/PD-1 pathway mediate immune suppression in malignant human brain tumors. Neuro-oncology.

[9] Fecci PE (2006). Increased regulatory T-cell fraction amidst a diminished CD4 compartment explains cellular immune defects in patients with malignant glioma. Cancer research.

[10] Batich KA, Swartz AM, Sampson JH (2015). Enhancing dendritic cell-based vaccination for highly aggressive glioblastoma. Expert opinion on biological therapy.

[11] Ashley DM (1997). Bone marrow-generated dendritic cells pulsed with tumor extracts or tumor RNA induce antitumor immunity against central nervous system tumors. The Journal of experimental medicine.

[12] Romani N (1994). Proliferating dendritic cell progenitors in human blood. The Journal of experimental medicine.

[13] Sallusto F, Lanzavecchia A (1994). Efficient presentation of soluble antigen by cultured human dendritic cells is maintained by granulocyte/macrophage colony-stimulating factor plus interleukin 4 and downregulated by tumor necrosis factor alpha. The Journal of experimental medicine.

[14] Nair, S., Archer, G. E. & Tedder, T. F. Isolation and generation of human dendritic cells. *Current protocols in immunology* Chapter 7, Unit732, 10.1002/0471142735.im0732s99 (2012). 10.1002/0471142735.im0732s99 PMC4559332 23129155

[15] Jonuleit H (1997). Pro-inflammatory cytokines and prostaglandins induce maturation of potent immunostimulatory dendritic cells under fetal calf serum-free conditions. European journal of immunology.

[16] Duarte S, Carle G, Faneca H, de Lima MC, Pierrefite-Carle V (2012). Suicide gene therapy in cancer: where do we stand now?. Cancer letters.

[17] Tomicic MT, Thust R, Kaina B (2002). Ganciclovir-induced apoptosis in HSV-1 thymidine kinase expressing cells: critical role of DNA breaks, Bcl-2 decline and caspase-9 activation. Oncogene.

[18] Chiocca EA, Rabkin SD (2014). Oncolytic viruses and their application to cancer immunotherapy. Cancer immunology research.

[19] Lawler SE, Speranza MC, Cho CF, Chiocca EA (2017). Oncolytic Viruses in Cancer Treatment: A Review. JAMA oncology.

[20] Melcher A, Parato K, Rooney CM, Bell JC (2011). Thunder and lightning: immunotherapy and oncolytic viruses collide. Molecular therapy: the journal of the American Society of Gene Therapy.

[21] Stupp R (2009). Effects of radiotherapy with concomitant and adjuvant temozolomide versus radiotherapy alone on survival in glioblastoma in a randomised phase III study: 5-year analysis of the EORTC-NCIC trial. The Lancet. Oncology.

[22] Kong DS (2017). Phase III randomized trial of autologous cytokine-induced killer cell immunotherapy for newly diagnosed glioblastoma in Korea. Oncotarget.

[23] Batich KA (2017). Long-term Survival in Glioblastoma with Cytomegalovirus pp65-Targeted Vaccination. Clinical cancer research: an official journal of the American Association for Cancer Research.

[24] Chang CN (2011). A phase I/II clinical trial investigating the adverse and therapeutic effects of a postoperative autologous dendritic cell tumor vaccine in patients with malignant glioma. Journal of clinical neuroscience: official journal of the Neurosurgical Society of Australasia.

[25] Cho DY (2012). Adjuvant immunotherapy with whole-cell lysate dendritic cells vaccine for glioblastoma multiforme: a phase II clinical trial. World neurosurgery.

[26] Jie X (2012). Clinical application of a dendritic cell vaccine raised against heat-shocked glioblastoma. Cell biochemistry and biophysics.

[27] Vik-Mo EO (2013). Therapeutic vaccination against autologous cancer stem cells with mRNA-transfected dendritic cells in patients with glioblastoma. Cancer immunology, immunotherapy: CII.

[28] Wheeler CJ, Das A, Liu G, Yu JS, Black KL (2004). Clinical responsiveness of glioblastoma multiforme to chemotherapy after vaccination. Clinical cancer research: an official journal of the American Association for Cancer Research.

[29] Yamanaka R (2005). Clinical evaluation of dendritic cell vaccination for patients with recurrent glioma: results of a clinical phase I/II trial. Clinical cancer research: an official journal of the American Association for Cancer Research.

[30] Yu JS (2004). Vaccination with tumor lysate-pulsed dendritic cells elicits antigen-specific, cytotoxic T-cells in patients with malignant glioma. Cancer research.

[31] Wheeler LA (2016). Phase II multicenter study of gene-mediated cytotoxic immunotherapy as adjuvant to surgical resection for newly diagnosed malignant glioma. Neuro-oncology.

[32] Westphal M (2013). Adenovirus-mediated gene therapy with sitimagene ceradenovec followed by intravenous ganciclovir for patients with operable high-grade glioma (ASPECT): a randomised, open-label, phase 3 trial. The Lancet. Oncology.

[33] Stragliotto G (2013). Effects of valganciclovir as an add-on therapy in patients with cytomegalovirus-positive glioblastoma: a randomized, double-blind, hypothesis-generating study. International journal of cancer.

[34] Rainov NG (2000). A phase III clinical evaluation of herpes simplex virus type 1 thymidine kinase and ganciclovir gene therapy as an adjuvant to surgical resection and radiation in adults with previously untreated glioblastoma multiforme. Human gene therapy.

[35] Su J (2016). Molecularly Targeted Drugs Plus Radiotherapy and Temozolomide Treatment for Newly Diagnosed Glioblastoma: A Meta-Analysis and Systematic Review. Oncology research.

[36] Li M (2017). The interventional effect of new drugs combined with the Stupp protocol on glioblastoma: A network meta-analysis. Clinical neurology and neurosurgery.

[37] Weller M (2017). Rindopepimut with temozolomide for patients with newly diagnosed, EGFRvIII-expressing glioblastoma (ACT IV): a randomised, double-blind, international phase 3 trial. The Lancet. Oncology.

[38] Cao JX (2014). Clinical efficacy of tumor antigen-pulsed DC treatment for high-grade glioma patients: evidence from a meta-analysis. PloS one.

[39] Artene SA (2016). Dendritic cell immunotherapy versus bevacizumab plus irinotecan in recurrent malignant glioma patients: a survival gain analysis. OncoTargets and therapy.

[40] Rampling R (2000). Toxicity evaluation of replication-competent herpes simplex virus (ICP 34.5 null mutant 1716) in patients with recurrent malignant glioma. Gene therapy.

[41] Mineta T, Rabkin SD, Yazaki T, Hunter WD, Martuza RL (1995). Attenuated multi-mutated herpes simplex virus-1 for the treatment of malignant gliomas. Nature medicine.

[42] Chiocca EA (2004). A phase I open-label, dose-escalation, multi-institutional trial of injection with an E1B-Attenuated adenovirus, ONYX-015, into the peritumoral region of recurrent malignant gliomas, in the adjuvant setting. Molecular therapy: the journal of the American Society of Gene Therapy.

[43] Csatary LK (2004). MTH-68/H oncolytic viral treatment in human high-grade gliomas. Journal of neuro-oncology.

[44] Shand N (1999). A phase 1-2 clinical trial of gene therapy for recurrent glioblastoma multiforme by tumor transduction with the herpes simplex thymidine kinase gene followed by ganciclovir. GLI328 European-Canadian Study Group. Human gene therapy.

[45] Immonen A (2004). AdvHSV-tk gene therapy with intravenous ganciclovir improves survival in human malignant glioma: a randomised, controlled study. Molecular therapy: the journal of the American Society of Gene Therapy.

[46] Soderberg-Naucler C, Johnsen JI (2015). Cytomegalovirus in human brain tumors: Role in pathogenesis and potential treatment options. World journal of experimental medicine.

[47] Fornara O (2016). Cytomegalovirus infection induces a stem cell phenotype in human primary glioblastoma cells: prognostic significance and biological impact. Cell death and differentiation.

[48] Borenstein M, Hedges LV, Higgins JP, Rothstein HR (2010). A basic introduction to fixed-effect and random-effects models for meta-analysis. Research synthesis methods.

[49] Tierney JF, Stewart LA, Ghersi D, Burdett S, Sydes MR (2007). Practical methods for incorporating summary time-to-event data into meta-analysis. Trials.

[50] Higgins JP, Thompson SG (2002). Quantifying heterogeneity in a meta-analysis. Statistics in medicine.

